# Psychometric properties and socio-demographic correlates of the Connor-Davidson Resilience Scale in three large population-based cohorts including Danish and Icelandic adults

**DOI:** 10.1016/j.xjmad.2025.100112

**Published:** 2025-02-17

**Authors:** Maria Didriksen, Hilda Daníelsdottir, Marín Dögg Bjarnadóttir, Cassie Overstreet, Karmel W. Choi, Lea Arregui Nordahl Christoffersen, Christina Mikkelsen, Thor Aspelund, Arna Hauksdóttir, Edda Bjork Thordardottir, Jóhanna Jakobsdóttir, Gunnar Tómasson, Christian Erikstrup, Bitten Aagaard, Mie T. Bruun, Henrik Ullum, Erik Sørensen, Ian C. Fischer, Robert H. Pietrzak, Joel Gelernter, Laura Campbell-Sills, Murray B. Stein, Thomas Werge, Ole B. Pedersen, Unnur Valdimarsdóttir, Sisse Rye Ostrowski, Andrew J. Schork

**Affiliations:** a Department of Clinical Immunology, Copenhagen University Hospital Rigshospitalet, Copenhagen, Denmark; b Department of Neuroscience, University of Copenhagen, Copenhagen, Denmark; c Centre of Public Health Sciences, Faculty of Medicine, University of Iceland, Reykjavík, Iceland; d Institute of Environmental Medicine, Karolinska Institutet, Stockholm, Sweden; e Department of Psychiatry, Yale School of Medicine, West Haven, CT, USA; f Department of Psychiatry, Massachusetts General Hospital, Boston, MA, USA; g Institute of Biological Psychiatry, Copenhagen Mental Health Services, Co-penhagen University Hospital, Copenhagen, Denmark; h Department of Clinical Immunology, Zealand University Hospital, Køge, Denmark; i Novo Nordisk Foundation Center for Basic Metabolic Research, Copenhagen University, Copenhagen, Denmark; j Department of Clinical Immunology, Aarhus University Hospital, Aarhus, Denmark; k Department of Clinical Medicine, Aarhus University, Aarhus, Denmark; l Department of Clinical Immunology, Aalborg University Hospital, Aalborg, Denmark; m Department of Clinical Immunology, Odense University Hospital, Odense, Denmark; n Statens Serum Institut, Copenhagen, Denmark; o US Department of Veterans Affairs National Center for Posttraumatic Stress Disorder, VA Connecticut Healthcare System, West Haven, CT, USA; p Department of Psychiatry, Yale School of Medicine, New Haven, CT, USA; q Department of Social and Behavioral Sciences, Yale School of Public Health, New Haven, CT, USA; r VA Connecticut Healthcare System, West Haven, CT, USA; s Departments of Psychiatry, Genetics, and Neuroscience, Yale University School of Medicine, New Haven, CT, USA; t Department of Psychiatry, University of California San Diego, La Jolla, CA, USA; u Department of Psychiatry and School of Public Health, University of California San Diego, La Jolla, CA, USA; v Institute of Biological Psychiatry, Copenhagen Mental Health Services, Copenhagen University Hospital, Copenhagen, Denmark; w Department of Clinical Medicine, Faculty of Health and Medical Sciences, University of Copenhagen, Copenhagen, Denmark; x Department of Epidemiology, Harvard TH Chan School of Public Health, Boston, MA, USA

## Abstract

Identifying specific factors affecting psychological resilience could be instrumental in developing new therapeutic strategies for improving and maintaining mental and physical health. To achieve this, an adequate measure of psychological resilience is essential. We studied the psychometric properties of the 10-item Connor-Davidson Resilience Scale (CD-RISC-10) across three population-based cohorts. The study included adult Danish individuals in the Danish Blood Donor Study (DBDS, N = 57,031), and the Icelandic cohorts Stress-And-Gene Analysis cohort (SAGA, N = 27,236), and COVID-19 National Resilience cohort (C19-Resilience, N = 20,373). Exploratory factor analysis revealed a consistent one-factor fit across cohorts. The scale demonstrated good internal consistency (Cronbach's alpha: 0.88–0.90) and longitudinal stability (Spearman correlation coefficients in DBDS: 0.71 across 1.5 years, 0.66 across two years). Mean CD-RISC-10 scores were consistent with previously reported scores in European populations. Lower scores were observed among participants with probable major depressive disorder, and higher scores were found with increasing age. Further evidence for construct validity was revealed as CD-RISC-10 scores moderated the association between financial trouble and depressive symptoms across all cohorts. Psychometric properties of the CD-RISC-10 were similar among women and men. Our findings support the use of the CD-RISC-10 as a reliable and valid unidimensional measure of psychological resilience in Danish and Icelandic populations.

## 1 Introduction

Psychological resilience refers to an individual's capacity to cope adaptively with adverse experiences. The COVID-19 pandemic underscored the critical role of resilience in mental health. Studies have shown that high psychological resilience mitigated the impact of pandemic-related stressors on psychological outcomes [1], [2]. It is a complex phenotype influenced by genetic [3], [4], [5] biological [6], [7], psychological [8], [9], and environmental factors [3], [10]. Identifying specific factors affecting psychological resilience could be instrumental in developing new therapeutic strategies for improving and maintaining mental and physical health. To achieve this, an adequate measure of psychological resilience is essential. However, creating a standardized measure to quantify this phenotype is challenging, particularly because people in different populations live in diverse contexts with varying cultures, socio-economic positions, exposure to stressors, and support systems. Consequently, populations have distinct cultural or societal norms that influence how individuals perceive and express resilience.

There are several ways of measuring psychological resilience. It can be operationalized as ‘observed’ resilience where the resilience phenotype applies to individuals exposed to some risk factor without developing the risk-related outcome (e.g., absence of PTSD symptoms following trauma exposure). This indirect way of conceptualizing or measuring resilience considers the impact of exposure to a stressor. Psychological resilience can also be measured directly using self-report tools of varying behaviors and cognitive patterns when exposed to challenges. In 2010, Connor and Davidson developed a self-report scale for measuring psychological resilience – the Connor-Davidson Resilience Scale (CD-RISC) [11]. The original 25-item scale (CD-RISC-25) assessed five factors, with factor 1 reflecting the notion of personal competence, high standards, and tenacity. Factor 2 describes trust in one’s instincts, tolerance of negative affect, and strengthening effects of stress. Factor 3 relates to the positive acceptance of change, and secure relationships. Factor 4 relates to control, and factor 5 to spiritual influences. The scale’s development focused on abilities that are important treatment outcome goals in anxiety, depression, and stress reactions. In the original validation study, the authors reported that the CD-RISC-25 demonstrated high internal consistency and good test-retest reliability. They also reported evidence of construct validity based on expected correlations with other phenotypes, such as perceived stress (negative) and hardiness (positive). Additionally, the scale’s validity was supported by observed variations in scores across groups hypothesized to have different levels of resilience, such as the general population versus patients with psychiatric disorders. Consequently, the developers suggested the CD-RISC-25 to be useful for conceptualizing resilience in biological studies and in clinical practice for identifying and nurturing personal qualities related to resilience [11]. However, subsequent factor analytical studies revealed limitations in the 25-item scale (e.g., unstable factor structure, redundant items), leading to the development of a shortened 10-item version (CD-RISC-10) [12]. Campbell-Sills and Stein (2007) demonstrated support for the internal consistency (Cronbach’s alpha = 0.85) [12], as well as construct validity of the CD-RISC-10 by showing that the composite score moderated the impact of retrospective reports of childhood maltreatment on adult psychiatric symptoms [10].

A point of debate has been whether psychological resilience is a stable trait or if it varies in individuals over time. Some studies have shown individual variations across age and during periods of changing life circumstances (such as getting a new job, completing an education etc.) or exposure to stressful life events (such as getting divorced, health problems etc.) [13]. Supporting this, the CD-RISC-25 score has been demonstrated to improve with treatment targeting aspects of resilience among individuals with post-traumatic stress disorder (PTSD) [11]. In line with the idea of psychological resilience being a dynamic process, Richardson et al. (1990) proposed that resilience is an accumulative construct, continuously building upon an individual’s previous coping experiences [14]. This perspective frames resilience as an effect of adverse exposures rather than a determinant of responses to them. Others have also suggested that psychological resilience may be defined as a dynamic interaction of protective factors, such as social support, family, and self-esteem [8]. If psychological resilience is indeed a dynamic characteristic that varies across the lifespan, knowledge about factors influencing such variations is important, as this may be useful both in understanding the phenotype and informing prevention and treatment strategies.

Based on prior studies of the scale, we hypothesized that the CD-RISC-10 would demonstrate a unidimensional structure with high internal consistency, and construct validity across cohorts. In the present study we examined the psychometric properties of the CD-RISC-10 in large and diverse general population samples from two different Nordic nationalities and following translation of the scale into Danish and Icelandic. Specifically, we investigated how CD-RISC-10 scores relate to sociodemographic characteristics and evaluated the scale’s psychometric properties in three independent cohorts consisting of individuals from Denmark (one cohort) and Iceland (two cohorts). These cohorts represent varied cultural norms, languages, and potential selection biases. Our study focused on assessing the factor structure, internal consistency, score stability over time, and construct validity of the CD-RISC-10, as well as identifying sociodemographic correlates of resilience scores. We compared our results internally, and to the previously published validation data based on CD-RISC-10 from Campbell-Sills and Stein [12].

## 2 Methods

### 2.1 The study cohorts

This study includes three adult population cohorts in Denmark and Iceland who responded to the CD-RISC-10; the Danish Blood Donor Study (DBDS, N = 57,031), and the Icelandic cohorts Stress-And-Gene Analysis cohort (SAGA, N = 27,236), and COVID-19 National Resilience cohort (C19-Resilience, N = 20,373). Psychological resilience was measured using the CD-RISC-10, translated into Danish or Icelandic as appropriate.

#### 2.1.1 The Danish blood donor study (DBDS)

DBDS [15] is a nationwide cohort leveraging Danish blood banks, with resilience data collected via questionnaires distributed in three phases (2021–2024), yielding 78,233 responses from 58,649 unique individuals (27,236 men and 31,413 women).

#### 2.1.2 The Stress-and-Gene Analysis Cohort (SAGA)

The SAGA cohort is an Icelandic cohort examining the health impact of psychological trauma among women. In total, 27,934 participants completed the CD-RISC-10 (2018–2019).

#### 2.1.3 COVID-19 national resilience cohort (C19-Resilience)

The COVID-19 National Resilience Cohort (C-19 Resilience) is a cohort initiated during the COVID-19 pandemic in Iceland. In total, 20,373 participants (12,722 women and 4730 men) completed the CD-RISC-10 (2020–2021).

### 2.2 Measures

Several self-report tools were applied in this study. Psychological resilience was measured using the CD-RISC-10, experience of depressive symptoms was measured using The Patient Health Questionnaire 9 item (PHQ9), and experience of financial troubles was estimated using two direct questions asking about this.

#### 2.2.1 Self-reported psychological resilience

CD-RISC-10 measures psychological resilience by focusing on the individual’s ability self-perceived to cope with adversities. The CD-RISC-10 consists of 10 items which are each rated on a 5-point Likert scale (0−4) ranging from 0: “not true at all” to 4: “true nearly all the time”. With the scoring based on summing of the items, the overall score range of CD-RISC-10 is 0–40 with a higher score indicating a higher psychological resilience level.

#### 2.2.2 Self-reported depressive symptoms

PHQ9 was used to measure the frequency of nine depressive symptoms within the past two weeks. Each item is rated on a 4-point Likert scale ranging from 0: “Not at all” to 3: “Nearly every day”. The total score ranges from 0 to 27. All PHQ9 symptoms are defined as present if respondents report experiencing them “More than half the days” or “Nearly every day”, except for the symptom assessing suicidal ideation, described as “Thoughts that you would be better off dead, or of hurting yourself,” which is defined as present if the respondent experiences this “Several days” or more often [16], [17]. Participants who reported at least five symptoms, of which one was either experiencing “Little interest or pleasure in doing things” or “Feeling down, depressed, or hopeless,” were classified as having probable major depressive disorder (pMDD) [16], [17].

#### 2.2.3 Self-reported financial troubles

Self-reported financial troubles were measured using similar questions across the three cohorts. In the DBDS cohort, individuals who responded “Yes, some” or “Yes a lot” to at least one of two questions asking if they had “experienced financial difficulties” or “had loss of income” within the last two years were considered to have been exposed to financial trouble. In the SAGA cohort, individuals who responded “Yes” to a question asking if they had “experienced severe financial loss or bankruptcy” within the last two years were considered exposed to financial trouble, whereas individuals from the C19-Resilience cohort were similarly classified if they responded “Fairly difficult” or “Very difficult” to a question asking “How easy or difficult has it been for you and your family (if applicable) to make ends meet financially in the past 12 months, i.e. to afford food, housing, and pay bills?”.

### 2.3 Statistical analyses

Overall, our aim was to determine whether the structure and function of the CD-RISC-10 scale varied within and between the three cohorts. This included clarifying variations between women and men and therefore, all analyses were repeated stratified by sex. In this study ‘sex’ refers to that recorded in the National population registers. To explore factor structure, stability in score over time, and construct validity including internal consistency several statistical tests were carried out.

All analyses were performed using R statistical software (v.4.3.0).

#### 2.3.1 Exploratory factor analysis

To test whether the CD-RISC-10 effectively measures psychological resilience as a singular dimension or as a composite of multiple latent dimensions within our cohorts, we conducted an exploratory factor analysis (EFA)*.* Even though we would expect a unidimensional structure, given variability in factor structures reported in the few prior studies, particularly in culturally distinct populations, we found an EFA suitable to evaluate whether CD-RISC-10 measures psychological resilience as a singular construct across our diverse cohorts without predefining its structure. Thus, in the initial phase of our study, we conducted Bartlett's test of sphericity and calculated the Kaiser-Meyer-Olkin (KMO) measure to assess if it was appropriate to conduct an EFA on CD-RISC-10 scale responses in each of the three cohorts. Bartlett's test was used to examine whether the intercorrelations between the items in the scale were statistically significant, a crucial precondition for factor analysis. Concurrently, the KMO measure evaluated the sampling adequacy by quantifying the proportion of shared variance among the items. Proceeding with EFA was considered appropriate with a significant result from Bartlett's test and a KMO value above 0.6. Such results would indicate both the presence of meaningful intercorrelations and sufficient shared variance among the CD-RISC-10 items. Subsequently, an EFA was conducted using the ‘psych’ [18] package in R Statistical software and applying ‘varimax’ rotation to improve interpretation and reduce likelihood of cross-loadings between potential factors. In addition, we tested whether findings would hold using “promax” rotation. To reduce the risk of overfitting, the decision regarding the number of factors to extract was made based on a parallel analysis including visual examination of a parallel analysis scree plots. We selected factors with eigenvalues surpassing those expected from random data. To complement the EFA, the reliability of the scale, was evaluated by calculating Cronbach's alpha, which indicates how well items on the scale measure the same construct.

In the DBDS, some participants had completed the CD-RISC-10 on multiple occasions. We included only the first-time responses of the CD-RISC-10 in the Bartlett’s test, KMO test, and EFA.

#### 2.3.2 Stability over time

Subsequently, for those in the DBDS cohort who had answered the CD-RISC-10 more than once, we calculated delta values to check the average change in the composite scores across time, and we assessed the stability of responses over time. Due to non-normal distribution of the scores, this was done by calculating Spearman correlation coefficient (SCC) to assess the within-individual rank correlation between the scores for the first and second response, and for those with three responses, for the first and third too. In total, 15,871 individuals had answered all 10 items on CD-RISC-10 twice. Of these, 31 individuals experienced a change of either more than −30 or + 30 in their score. We believed this to be unlikely. These individuals were excluded from this analysis. Of the remaining 15,840 individuals, 2358 answered the scale three times.

#### 2.3.3 Construct validity

Exposures that requires considerable adjustment of individuals’ usual activities, such as financial trouble, are associated with an increased risk of experiencing depressive symptoms [8], [19]. Therefore, we estimated the construct validity of the CD-RISC-10 in all three cohorts by testing whether CD-RISC-10 scores moderated the impact of financial trouble on the severity of depressive symptoms (total PHQ9 score) [17].

The construct validity was tested using a hierarchical linear regression approach with ‘financial trouble’ included as the independent variable and ‘PHQ-9 scores’ as the dependent on the first step and adding an interaction term (‘financial trouble’ × ‘CD-RISC-10 scores’) in the second.

## 3 Results

Table 1 presents the sociodemographic and economic characteristics of the three cohorts (DBDS, SAGA, and C19-Resilience) and their associations with CD-RISC-10 scores.

**Table 1:** Baseline Characteristics of the study population.

***Cohort***			**DBDS**				**SAGA**				**C19-Resilience**				
			**Total**	**CD-RISC-10 total**			**Total**	**CD-RISC-10 total**				**Total**	**CD-RISC-10 total**		
			N (%)	Mean (SD)	P value*	Cohens *d* (95% CI)**	N (%)	Mean (SD)	P value	Cohens *d* (95% CI)**	N (%)		Mean (SD)	P value	Cohens *d* (95% CI)**
**Total**			58,649	31.2 (5.9)			27,934					20,373			
**Sex**					<0.0001	-0.18 (-0.20; -0.17)								<0.0001	
	Women		31,413 (53.6)	30.7 (6.0)			27,934 (100)	27.3 (7.5)				12,722 (72.9)	30.6 (6.4)		0.16 (0.13; 0.19)
	Men		27,236 (46.4)	31.8 (5.8)		*Reference*	0	-				4730 (27.1)	31.7 (5.8)		*Reference*
**Women**															
**Age groups**					<0.0001				<0.0001				<0.0001		
	18-29 years old		6379 (19.3)	29.7 (5.9)		-0.40 (-0.44; -0.36)	5308 (19.0)	24.8 (7.9)		-0.51 (-0.55; -0.47)		998 (4.2)	27.4 (7.7)		-0.53 (-0.61; -0.46)
	30-39 years old		6168 (18.7)	30.2 (6.0)		-0.27 (-0.30; -0.23)	5633 (20.2)	26.8 (7.6)		-0.26 (-0.30; -0.22)		1642 (12.9)	29.4 (6.9)		-0.30 (-0.35; -0.25)
	40-49 years old		7389 (22.4)	31.0 (5.9)		-0.12 (-0.16; -0.09)	6216 (22.3)	27.7 (7.4)		-0.14 (-0.18; -0.09)		2832 (22.3)	30.8 (6.7)		-0.08 (-0.12; -0.04)
	50-59 years old		7947 (24.1)	31.4 (5.9)		-0.03 (-0.06; 0.00)	6417 (23.0)	28.4 (7.3)		-0.04 (-0.08; -0.00)		3806 (29.9)	31.4 (6.1)		0.03 (0.00; 0.06)
	≥60 years old		5100 (15.5)	31.4 (6.0)		*Reference*	4360 (15.6)	28.7 (7.0)		*Reference*		3444 (27.1)	31.2 (5.8)		*Reference*
**Current depressive symptoms**					<0.0001				<0.0001					<0.0001	
	Yes		851 (4.8)	24.9 (7.4)		-1.04 (-1.11; -0.97)	7530 (27.0)	22.1 (7.5)		-1.17 (-1.20; -1.14)		2531 (19.9)	25.4 (7.5)		-0.98 (-1.02; -0.93)
	No		17,062 (95.2)	31.1 (5.9)		*Reference*	18,879 (65.4)	29.8 (6.2)		*Reference*		9,777 (76.9)	32.1 (5.4)		*Reference*
	Unknown		12,353	30.6 (5.7)		-0.09 (-0.11; -0.06)	2125 (7.6)	23.9 (7.5)		-0.92 (-0.97; -0.88)		414 (3.3)	27.0 (7.3)		-0.73 (-0.83; -0.64)
**Recent financial trouble**					<0.0001				<0.0001					<0.0001	
	Yes	1068 (6.2)		29.5 (6.9)		-0.23 (-0.29; -0.17)	3320 (11.9)	26.1 (8.0)		-0.25 (-0.28; -0.21)		1750 (13.8)	27.1 (7.6)		-0.61 (-0.67; -0.56)
	No	16,845 (94.0)		30.9 (6.1)		*Reference*	11022 (39.5)	28.0 (7.3)		*Reference*		10,907 (85.7)	31.2 (6.1)		*Reference*
	Unknown	13,500		30.6 (5.7)		-0.05 (-0.07; -0.03)	13,592 (48.7)	26.9 (7.6)		-0.15 (-0.17; -0.13)		65 (0.05)	29.1 (8.8)		-0.28 (-0.52; -0.05)
**Educational level×**				0.311					<0.0001					<0.0001	
	Primary		1221 (5.49)	30.9 (6.9)		*Reference*	4099 (14.7)	24.2 (8.3)		*Reference*		1677 (13.2)	28.3 (7.4)		*Reference*
	Secondary		9117 (41.0)	30.8 (6.5)		-0.01 (-0.08; 0.04)	8629 (30.9)	26.2 (7.7)		0.25 (0.22; 0.28)		3247 (25.5)	29.6 (6.9)		0.18 (0.13; 0.23)
	Tertiary A (BSc or equivalent)		7030 (31.6)	30.9 (6.1)		0 (-0.06; 0.06)	8832 (31.6)	28.0 (7.0)		0.48 (0.45; 0.51)		4477 (35.2)	31.2 (6.1)		0.43 (0.38; 0.47)
	Tertiary B (MSc or above)		4832 (21.7)	30.9 (6.0)		0 (.0.06; 0.06)	6254 (22.4)	29.8 (6.5)		0.77 (0.73; 0.81)		3269 (25.7)	32.2 (5.6)		0.60 (0.55; 0.64)
	Unknown		37 (0.17)	31.2 (7.9)		0.04 (-0.28; 0.37)	120 (0.4)	23.3 (8.1)		-0.11 (-0.29; 0.07)		52 (0.04)	27.6 (8.6)		-0.09 (-0.30; 0.12)
**Personal monthly income×**					<0.0001				<0.0001					<0.0001	
	Low		8038 (36.1)	30.0 (6.6)		*Reference*	8348 (29.9)	24.4 (8.0)		*Reference*		2460 (19.3)	27.6 (7.40)		*Reference*
	Medium		8700 (39.1)	30.8 (6.2)		0.13 (0.09; 0.16)	14,749 (52.8)	28.0 (7.0)		0.48 (0.45; 0.50)		6855 (53.9)	30.7 (6.2)		0.46 (0.42; 0.50)
	High		5268 (23.7)	32.2 (5.6)		0.35 (0.32; 0.39)	3725 (13.3)	31.1 (6.1)		0.86 (0.82; 0.91)		2795 (22.0)	33.3 (5.1)		0.85 (0.79; 0.91)
	Unknown		231 (1.04)	30.4 (7.0)		0.06 (-0.07; 0.19)	1112 (4.0)	26.5 (8.0)		0.26 (0.21; 0.32)		612 (4.8)	30.6 (6.6)		0.41 (0.33; 0.49)
**Civil status×**					<0.0001				<0.0001					<0.0001	
	Married/in relationship		11,764 (52.9)	31.2 (6.1)		*Reference*	21,047 (75.3)	27.6 (7.4)		*Reference*		9575 (75.3)	31.0 (6.3)		*Reference*
	Single/divorced /windowed		10,473 (47.1)	30.4 (6.4)		-0.13 (-0.15; -0.19)	6732 (24.1)	26.3 (7.9)		-0.17 (-0.20; -0.15)		3100 (24.4)	29.7 (7.1)		-0.21 (-0.24; -0.17)
	Unknown		-	-		-	155 (0.6)	24.3 (8.5)		-0.45 (-0.61; -0.29)		47 (0.03)	27.6 (7.6)		-0.51 (-0.75; -0.28)
**Men**															
**Age groups**					<0.0001									<0.0001	
	18-29 years old		3790 (13.2)	31.2 (5.5)		-0.33 (-0.26; -0.40)						200 (4.22)	28.3 (7.3)		-0.62 (-0.77; -0.47)
	30-39 years old		5210 (18.1)	31.1 (5.7)		-0.28 (-0.34, -0.23)						291 (6.15)	30.0 (6.3)		-0.33 (-0.45; -0.21)
	40-49 years old		6451 (22.4)	31.5 (5.7)		-0.16 (-0.21; -0.12)						812 (17.2)	31.5 (6.4)		-0.07 (-0.15; 0.01)
	50-59 years old		7673 (26.7)	32.2 (5.7)		-0.03 (-0.07; 0.01)						1529 (32.3)	32.2 (5.6)		0.05 (-0.02; 0.12)
	≥60 years old		5620 (19.6)	32.4 (5.8)		*Reference*						1898 (40.1)	31.9 (5.6)		*Reference*
**Current depressive symptoms**				<0.0001										<0.0001	
	Yes		442 (3.0)	24.9 (7.7)		-1.07 (-1.14; -1.00)						538 (11.4)	25.8 (7.5)		-1.08 (-1.15; -1.01)
	No		14,5,28 (97.0)	32.1 (5.7)		*Reference*						4070 (86.0)	32.6 (5.1)		*Reference*
	Unknown		11,229	31.7 (5.5)		-0.07 (-0.09; -0.05)						122 (2.4)	26.7 (7.1)		-0.93 (-0.93; -0.77)
**Recent financial trouble**					<0.0001									<0.0001	
	Yes		921 (6.2)	31.0 (6.7)		-0.15 (-0.20; -0.09)						423 (8.9)	28.3 (7.4)		-0.58 (-0.65; -0.51)
	No		14,049 (93.8)	31.9 (5.9)		*Reference*						4293 (90.7)	32.0 (5.6)		*Reference*
	Unknown		12,266	31.7 (5.5)		-0.03 (-0.05; -0.01)						14 (0.03)	31.4 (6.9)		-0.09 (-0.42; 0.23)
**Educational level×**					0.674									<0.0001	
	Primary		1284 (6.80)	31.7 (6.9)		*Reference*						560 (11.8)	29.8 (6.9)		*Reference*
	Secondary		9384 (49.7)	32.0 (6.3)		0.05 (0.00; 0.09)						1842 (38.9)	31.3 (6.0)		0.23 (0.18; 0.28)
	Tertiary A (BSc or equivalent)		3804 (20.1)	32.0 (5.8)		0.05 (0.00; 0.09)						1290 (27.3)	31.9 (5.5)		0.35 (0.30; 0.40)
	Tertiary B (MSc or above)		4362 (23.1)	31.8 (5.8)		0.01 (-0.03; 0.06)						1004 (21.2)	33.0 (5.3)		0.49 (0.43; 0.55)
	Unknown		46 (0.24)	31.9 (8.0)		0.03 (-0.20; 0.25)						34 (0.07)	29.5 (7.30)		-0.04 (-0.30; 0.21)
**Personal monthly income×**					<0.001										<0.0001
	Low		5543 (29.4)	31.5 (6.6)		*Reference*						362 (7.7)	28.7 (7.9)		*Reference*
	Medium		4880 (25.8)	31.3 (6.4)		-0.03 (-0.07; 0.01)						1857 (39.3)	30.7 (6.2)		0.29 (0.24; 0.34)
	High		8312 (44.0)	32.6 (5.6)		0.18 (0.14; 0.21)						2316 (49.0)	33.0 (4.9)		0.62 (0.57; 0.67)
	Unknown		-	-								195 (4.1)	31.5 (5.6)		0.38 (0.23; 0.54)
**Civil status×**					<0.0001									<0.0001	
	Married/in relationship		12,221 (64.7)	32.3 (5.9)		*Reference*						3982 (84.2)	32.0 (5.6)		*Reference*
	Single/divorced windowed		6659 (35.3)	31.3 (6.5)		-0.16 -0.18; -0.13)						3100 (65.5)	29.9 (7.1)		-0.34 (-0.37; -0.30)
	Unknown		-	-		-						47 (0.09)	28.9 (6.8)		-0.48 (-0.74; -0.23)

*Calculated using the non-parametric Kruskal-Wallis test or Mann-Whitney U test dependent on number of categories

× Information on self-assessed current depressive symptoms, educational level, income, and civil status was only available for a sub-group of the DBDS cohort, the “unknown” category describes those for whom the information should have been available for.

**For variables with more than two categories, Cohens *d* pair-wise estimates are reported versus the same reference group. The reference groups were, for ‘Age groups’: ≥60 years old, for ‘Educational level’: Primary, and for ‘Personal monthly income’: Low

In terms of demographic characteristics, women showed modestly lower CD-RISC-10 scores compared to men in both DBDS (mean difference = −1.10, SEM (standard error of the mean) = 0.05, t = -20.9, P < 0.0001) and C19-Resilience cohorts (mean difference = −1.03, SEM = 0.10, t = -9.88, P < 0.0001). Across all cohorts, CD-RISC-10 scores increased with age for both sexes. Among women, the age effect was strongest in SAGA (β (linear regression coefficient) = 0.10 per year, SE = 0.003, P < 0.0001), followed by C19-Resilience (β = 0.08, SE = 0.005, P < 0.0001) and DBDS (β = 0.05, SE = 0.002, P < 0.0001). Men showed similar but slightly lower age effects in both DBDS (β = 0.04, SE = 0.003, P < 0.0001) and C19-Resilience (β = 0.06, SE = 0.008, P < 0.0001).

Current pMDD and financial status showed strong associations with resilience scores. Participants reporting current depressive symptoms had significantly lower CD-RISC-10 scores across all cohorts, with mean differences in CD-RISC-10 scores ranging from 6.2 to 7.7 points (all P < 0.0001). Similarly, those reporting financial troubles showed lower resilience scores, with the effect being most pronounced in the C19-Resilience cohort (women: mean difference = 4.1, SEM = 0.19; men: mean difference = 3.7, SEM = 0.37; both P < 0.0001) compared to DBDS (women: mean difference = 1.5, SEM = 0.21; men: mean difference = 0.9, SEM = 0.22) and SAGA cohorts (women: mean difference = 1.9, SEM = 0.15).

Socioeconomic factors demonstrated varying relationships with resilience across cohorts. Higher educational level was associated with higher CD-RISC-10 scores in general in SAGA and C19-Resilience cohorts but not in DBDS. Higher income levels, however, were consistently associated with higher CD-RISC-10 scores across all cohorts. The effect was most pronounced among women, with mean increases from low to high income categories of 6.7 points (SEM = 0.13) in SAGA and 5.7 points (SEM = 0.18) in C19-Resilience, compared to 2.2 points (SEM = 0.11) in DBDS (all P < 0.0001).

Relationship status was also associated with resilience scores, with participants in relationships showing higher CD-RISC-10 scores compared to single participants. This effect was strongest among men in the C19-Resilience cohort (mean difference = 2.1, SEM = 0.16, P < 0.0001) and more modest but consistent across other cohorts and genders (mean differences ranging from 0.8 to 1.3 points, all P < 0.0001).

The magnitude of these associations remained relatively consistent across cohorts, though the C19-Resilience cohort generally showed stronger effects for socioeconomic factors compared to DBDS and SAGA cohorts. In addition, Cohen’s d estimates, presented in Table 1, indicate the magnitude of differences in CD-RISC-10 scores between key groups. For example, individuals with depressive symptoms reported substantially lower resilience, with their mean score being between 0.98 (SAGA) to 1.17 (DBDS) standard deviations below that of individuals without depressive symptoms. This reflects large and clinically meaningful differences. Similarly, resilience scores were moderately lower among participants with recent financial troubles (Cohen’s d = −0.23 to −0.61). These findings underscore the scale’s ability to differentiate between populations with varying psychological and socioeconomic challenges.

*Kaiser-Meyer-Olkin analysis and Bartlett’s test of sphericity* The analysis revealed an overall measure of sampling adequacy (MSA) of 0.93 in the DBDS cohort, 0.94 in the SAGA cohort, and 0.93 in C19-Resilience cohort. This suggests a high level of common variance among the items. The Bartlett's test of sphericity was statistically significant in all cohorts (DBDS: Bartlett's K-squared = 14101, df = 9, P < 2.2 ×10^−16^; SAGA: Bartlett's K-squared 2726.2, df = 9, P < 2.2 ×10^−16^; C19-Resilience: Bartlett's K-squared = 5140.5, df = 9, P < 2.2 ×10^−16^), indicating that the correlation matrix is not an identity matrix, and that there are correlations among a least some of the items.

Sex-stratified analyses showed similar results with MSA, women = 0.93/men = 0.93 in DBDS, and MSA, women = 0.93/Men = 0.93 in C19-Resilience. The Bartlett's test of sphericity was statistically significant among both men and women (DBDS, women: Bartlett's K-squared = 8010.5, df = 9, P < 2.2 ×10^−16^, men: Bartlett's K-squared = 6102.1, df = 9, P < 2.2 ×10^−16^; C19-Resilience, women: Bartlett's K-squared = 3475.9, df = 9, P < 2.2 ×10–16, men: Bartlett's K-squared = 1730.9, df = 9, P < 2.2 ×10–16). Thus, both the KMO test and Bartlett’s test of sphericity suggest that the CD-RISC-10 scale is suitable for factor analysis in all three cohorts, in both sexes, combined and separately.

### 3.1 Exploratory factor analysis of the CD-RISC-10 scale

**Fig. 1:**
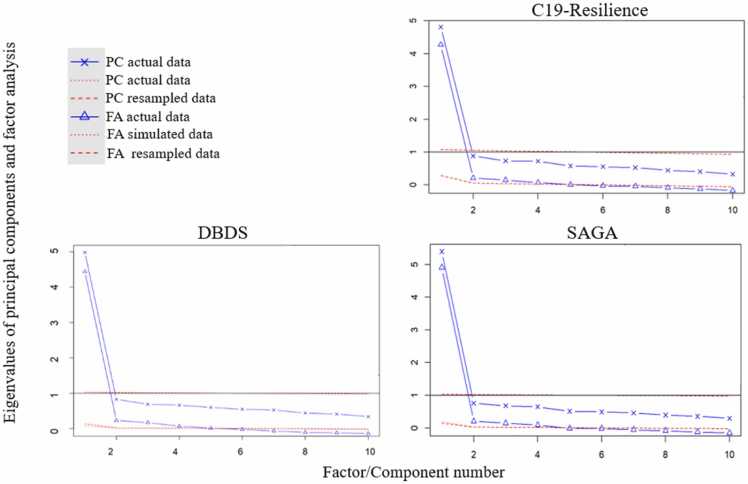
Parallel Scree Plots displaying eigenvalues for CD-RISC-10.

To identify number of factors to extract, we conducted a parallel analysis including visualization by a scree plot in each cohort (Fig. 1, sex-stratified parallel analysis scree plots are displayed in Supplemental materials, Figure S1). The eigenvalues demonstrate the amount of variance explained by each factor relative to the total variance. Since the eigenvalues when exceeding one factor are < 1, we concluded that handling the 10-item questionnaire as a unidimensional scale is the most appropriate. For the one-factor solution it seems that all items strongly correlate with a single underlying factor – psychological resilience, as the loadings varies between 0.56 and 0.78 in DBDS, between 0.61 and 0.80 in SAGA, and between 0.54 and 0.79 in C19-Resilience. In a previous analysis by Campbell-Sills and Stein [12], comprising 1743 undergraduates from San Diego State University, USA, (SDSU) the loadings varied between 0.44 and 0.74 [12] (in general factor loadings>0.30 usually indicates moderate correlation between the item and the factor) (Table 2). The one-factor solution explains 44 % of the total variance in DBDS, 49 % in SAGA, and 45 % in C19-Resilience.

**Table 2:** Displays factor loadings representing correlation between each item and factor(s) extracted from the data.

			One-factor solution factor loadings				
			**DBDS**	**SAGA**	**C19-Resilience**		**SDSU**^12^*
Item		Description					
1	Able to adapt to change		0.56	0.66		0.65	0.44
2	Can deal with whatever comes		0.72	0.76		0.73	0.72
3	Tries to see the humorous side of problems		0.63	0.61		0.54	0.46
4	Gets stronger when coping with stress		0.63	0.61		0.60	0.58
5	Tend to bounce back after illness		0.61	0.62		0.59	0.61
6	Can achieve goals despite obstacles		0.66	0.75		0.74	0.63
7	Can stay focused under pressure		0.71	0.74		0.73	0.62
8	Not easily discouraged by failure		0.65	0.67		0.61	0.63
9	Thinks of self as strong person		0.78	0.80		0.79	0.74
10	Can handle unpleasant feelings		0.69	0.75		0.72	0.57

The factor loadings were robust when applying promax rotation.

* Original validation of the CD-RISC-10 in N = 1743 undergraduates from San Diego University, US12

#### 3.1.1 Sex stratified analyses

The sex-stratified EFA showed similar results as the full-sample EFA, with marginal differences in factor loadings between sexes. For women in DBDS: TLI = 0.916, RMSEA = 0.086 (90 % CI: 0.085–0.088), BIC, 1-factor = 7656.3, and diagonal values = 0.99; for men in DBDS: TLI = 0.939, RMSEA = 0.073 (90 % CI: 0.071–0.075), BIC = 4580.85 = 2625, and based on diagonal values = 0.99. For women in C19-Resilience: TLI = 0.93, RMSEA = 0.082 (90 % CI: 0.08 – 0.085), diagonal values = 0.99; for men in C19-Resilience: TLI = 0.94 RMSEA = 0.07 (90 % CI: 0.066 – 0.074), BIC = 550.04, and based on diagonal values = 0.99.

Among both women and men, the one-factor solution explained 44 % of the total variance in DBDS, whereas 46 % and 43 % was explained among women and men in C19-resilience, respectively.(Table 3)

**Table 3:** Displays sex-stratified factor loadings representing correlation between each item and factor(s) extracted from the data.

			**DBDS**		**C19-Resilience**		**SAGA**
			Women	Men	Women	Men	Women
Item	Description						
1		Able to adapt to change	0.56	0.55	0.66	0.64	0.66
2		Can deal with whatever comes	0.72	0.71	0.74	0.73	0.76
3		Tries to see the humorous side of problems	0.64	0.63	0.55	0.49	0.61
4		Gets stronger when coping with stress	0.62	0.63	0.60	0.57	0.61
5		Tend to bounce back after illness	0.62	0.60	0.60	0.55	0.62
6		Can achieve goals despite obstacles	0.65	0.67	0.74	0.73	0.75
7		Can stay focused under pressure	0.70	0.71	0.74	0.71	0.74
8		Not easily discouraged by failure	0.65	0.64	0.62	0.55	0.67
9		Thinks of self as strong person	0.78	0.78	0.79	0.80	0.80
10		Can handle unpleasant feelings	0.70	0.68	0.73	0.69	0.75

### 3.2 Stability over time

The score stability over time was only examined in the DBDS cohort, as this was the only cohort with longitudinal data on the scale. In total, 15,840 individuals had answered all 10 items on CD-RISC-10 at least two times.

**Fig. 2:**
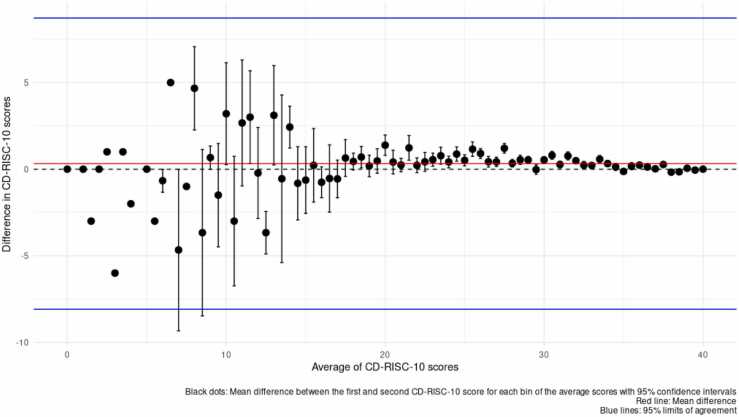
Bland-Altman plot displaying test-retest stability between first and second CD-RISC-10 scores.

The median change in CD-RISC-10 score between first and second response was delta= 0 (IQR: −2; +2), while the mean change was delta= +0.32 (standard deviation (SD) = 4.29) and the median time interval between answers was 389 days (IQR: 384;509). The Spearman Correlation Coefficient (SCC) between first and second CD-RISC-10 scores was 0.71, P value< 0.001. The test-retest stability was also assessed using a Bland-Altman plot (Fig. 2). The plot showed good agreement between the first and second scores, with a mean difference close to 0 and most differences falling within the 95 % limits of agreement.

A total of 2358 individuals answered a third time where we observed a median change in score of 0 (IQR: −3;3) and a mean of 0.02 (SD=4.42) between first and third response. The median number of days between the first and third response was 836 days (IQR: 735;939). SCC between first and last scores was 0.66, P-value< 0.001. CD-RISC-10 scores were stable over multiple administrations across multiple years, although there were slightly positive trends with time consistent with increased scores in later ages.

In total, 8318 women completed the CD-RISC-10 at least twice, whereas this was the case for 7522 men. Of these, 1182 women and 1176 men answered the scale three times. Time intervals between the answers were similar as to the combined cohort.

For women (N = 8318), the median change in CD-RISC-10 score between first and second assessment was delta= 0 (IQR: −2; +3), while the mean change + 0.43 (SD = 4.43) and the median time interval between answers was 389 days (IQR: 384;509). SCC between first and second CD-RISC-10 scores was 0.71, P value< 0.001. Between first and third assessment (N = 1182), delta = 0 (IQR: −3; +3), mean change = + 0.06 (SD = 4.60), and SCC = 0.65, P < 0.001. For men, the median change between first and second assessment was 0 (IQR: −2; 2) with a mean change of 0.20 (SD = 4.12), SCC = 0.71, P < 0.001. Between their first and third assessment (N = 1176) we observed median = 0 (IQR: −3; +2), mean = -0.02 (SD = 4.27), SCC = 0.66, P < 0.001.

### 3.3 Reliability and construct validity

The internal consistency of the CD-RISC-10 was assessed by calculating Cronbach’s alpha. The alpha value was estimated to be 0.89 in DBDS, 0.90 in SAGA, and 0.88 in C19-Resilience, which indicates good internal consistency and reliability, suggesting that the CD-RISC-10 items are measuring the same overall construct and that several administrations of the CD-RISC-10 scale will result in a consistent composite score. The item-total correlations ranged from 0.33 to 0.67 in DBDS, 0.36–0.67 in SAGA, and 0.30–0.64 in C19-Resilience.

To assess construct validity recent financial trouble was used as a risk exposure for depressive symptoms. In total, 67,510 individuals (DBDS, N = 32,883, SAGA, N = 14,342, C19-Resilience, N = 20,285) had complete data on the CD-RISC-10, questions on financial trouble, and the PHQ9 [16].

**Fig. 3:**
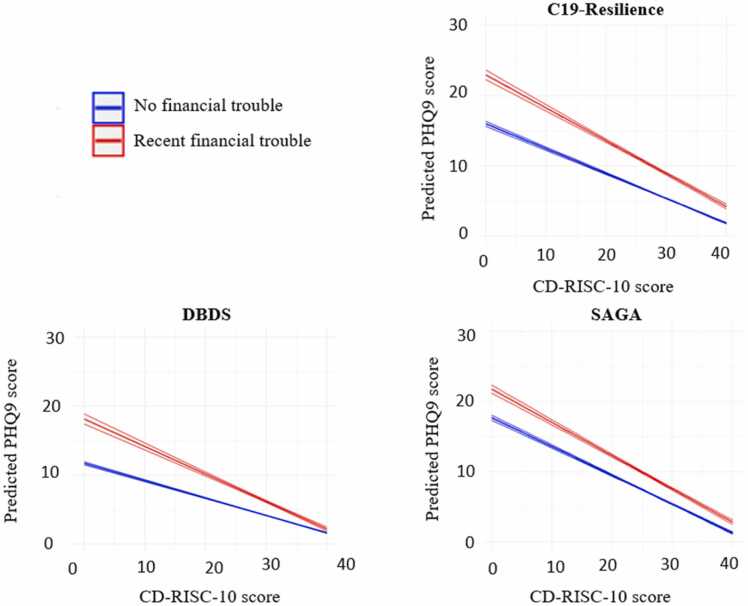
Effect of CD-RISC-10 scores in moderating the association between financial trouble and PHQ-9 scores.

A total of 7729 individuals reported recent financial trouble at the time of assessment (DBDS, N = 2025, SAGA, N = 3320, C19-Resilience, N = 2384). A hierarchical linear regression was conducted with Step 1 being a simple linear model including PHQ-9 scores as the dependent variable and recent financial trouble as the independent variable. This model revealed higher PHQ-9 scores in those reporting financial trouble (DBDS (sexes combined): β, financial trouble = 2.33, SE = 0.09, t = 24.7, P < 0.0001; SAGA (only women): β, financial trouble = 3.17, SE = 0.12, t = 27.0, P < 0.0001, C19-Resilience (sexes combined) β, financial trouble = 5.26, SE = 0.12, t = 45.9, P < 0.0001). Step 2 being the main effects model included PHQ-9 scores as the dependent variable and both ‘recent financial trouble’ and the CD-RISC-10 score as independent variables. In the Step 2 model, ‘recent financial trouble’ and CD-RISC-10 were positively and negatively associated with level of experienced depressive symptoms (PHQ9 score), respectively, across all cohorts (DBDS (sexes combined) β, financial trouble = 1.99, SE = 0.09, t = 23.0, P < 0.0001, β, CD-RISC-10 = -0.26, SE = 0.003, t = -77.6, P < 0.0001; SAGA (only women): β, financial trouble = 2.36, SE = 0.10, t = 24.2, P < 0.0001, β, CD-RISC-10 = -0.43, SE = 0.01, t = -77.6, P < 0.0001; C19-Resilience (sexes combined) β, financial trouble = 3.74, SE = 0.10, t = 36.3, P < 0.0001, β, CD-RISC-10 = -0.38, SE = 0.01, t = -70.4, P < 0.0001). Step 3 (the interaction model) added the ‘recent financial trouble’ × CD-RISC-10 interaction. In the Step 3 model, the interaction term was statistically significant (DBDS (sexes combined): β = -0.15, SE = 0.003, t = -77.6 P < 0.0001; SAGA (only women): β = -0.06, SE = 0.01, t = -5.11, P < 0.0001, C19-Resilience (sexes combined): β = -0.11, SE = 0.01, t = -8.31, P < 0.0001) supporting both the moderation hypothesis and the construct validity of the scale (Table 4, Fig. 3 and S2). When estimating predicted PHQ-9 scores based on CD-RISC-10 score and experience of financial trouble it was evident that the expected PHQ-9 scores became more similar between those with and without recent financial trouble, as the CD-RISC-10 score increased (Fig. 3). The same moderation effect was observed in sex-stratified analyses (Table 4, and Fig. 3 and S2). Observed PHQ-9 and CD-RISC-10 scores stratified by experience of recent financial troubles are displayed in Figures S3 and S4.

**Table 4:** Hierarchical linear regression models.

			**DBDS**		**SAGA**		**C19-Resilience**	
Model step*			**β**	**P value**	**β**	**P value**	**β**	**P value**
**All combined**								
1)	Financial trouble		2.33	< 0.001			5.26	< 0.0001
2)	CD-RISC−10 score		−0.26	< 0.001			−0.38	< 0.0001
3)	Interaction term		−0.15	< 0.001			−0.11	< 0.0001
**Women**								
1)	Financial trouble		2.73	< 0.001	3.17	< 0.0001	6.29	< 0.0001
2)	CD-RISC−10 score		−0.27	< 0.001	−0.43	< 0.0001	−0.36	< 0.0001
3)	Interaction term		−0.14	< 0.001	−0.06	< 0.001	−0.10	< 0.0001
**Men**								
1)	Financial trouble		3.26	< 0.001			9.43	< 0.0001
2)	CD-RISC−10 score		−0.24	< 0.001			−0.31	< 0.0001
3)	Interaction term		−0.15	< 0.001			−0.19	< 0.0001

* Model step 1) PHQ9 score ∼ financial trouble

Model step 2) PHQ9 score ∼ financial trouble + CD-RISC-10 score

Model step 3) PHQ9 score ∼ financial trouble + CD-RISC-10 score + financial trouble:CD-RISC-10 score PHQ9 score = patient health questionnaire 9-items, used to assess experience of depressive symptoms CD-RISC-10 score = Connor-Davidson Resilience scale score

OBS: estimates change marginally when adjusted for age (and sex in the combined model)

## 4 Discussion

Based on this comprehensive examination of the CD-RISC-10's psychometric properties and associated sociodemographic and economic characteristics across three Nordic cohorts: The DBDS (in Danish), SAGA, and C19-Resilience (in Icelandic), this study demonstrate that the CD-RISC-10 exhibits sound psychometric properties across these populations, with good internal consistency, high score stability over time, and construct validity. The scale maintains a comparable unidimensional factor structure across all three cohorts. This is supported by high Cronbach's alpha estimates, suggesting all 10 items consistently measure the same underlying construct. Therefore, we recommend treating the scale as unidimensional and calculating an overall score based on all 10 items, which aligns with recommendations made in previous studies based on both general population samples as well as patient samples, and that reported Cronbach’s alphas ranging from 0.80 to 0.90 [12], [20], [21], [22], [23], [24], [25], [26]. These findings collectively indicate that the scale is consistent across these contextually and culturally different populations.

Despite the potential for self-reported psychological resilience to be influenced by cultural, contextual, and psychological factors, we observed substantial consistency in the properties of the CD-RISC-10 across the cohorts. Worth noting though is that Denmark and Iceland have quite similar cultural and social systems. The variations noted could plausibly be attributed to linguistic differences between the Danish and Icelandic versions, rather than substantial cultural divergences. We observed that the DBDS and C19-Resilience cohorts displayed somewhat higher mean CD-RISC-10 scores than the SAGA cohort. This could be partly explained by selection in DBDS and C19-Resilience, which were based on voluntary active participation, potentially overrepresenting individuals with high psychological resilience. Part of the difference in mean scores may also be because both of these cohorts included women and men, whereas SAGA only included women (and we observe a tendency for higher scores among men). Mean CD-RISC-10 scores in our study (ranging from 27.2 to 33.5) align with other population-based cohorts of European descent, in particular a study including 1922 Spanish adults (aged 18–60) with a mean CD-RISC-10 score of 29.0 (0.1) [27]. Our findings support the notion that psychological resilience is normative in the general population, meaning reporting low resilience appears to be the exception. A caveat is that few studies have examined the CD-RISC-10 in general adult European populations. Most have focused on specific patient groups, students, or individuals working in highly stressful environments such as healthcare employees and military soldiers [12], [21], [22], [23], [25], [26], [28].

The nature of psychological resilience remains a subject of debate, with discussions centering on whether it represents a cumulative process, an independent trait, or merely the absence of risk exposure. Some have suggested that psychological resilience represents an accumulative phenomenon contingent on previous experiences with positive or negative adaption to adverse exposures, which raises the question of potential misclassification bias in individuals without prior adverse exposures. We observed lower CD-RISC-10 scores among participants with pMDD and we observed a negative association between CD-RISC-10 score and experience of depressive symptoms (PHQ9 score), corroborating previous findings that the scale effectively differentiates between general population and patient groups [20]. Additionally, we found higher mean scores among men compared to women, and a slight increase in mean scores as a function of increasing age. This trend of increase across ages could support the idea of psychological resilience as a cumulative construct, suggesting that individuals may become more resilient as they overcome life's obstacles. However, it could also indicate that people with lower resilience are less likely to reach higher age, and therefore are differentially censored out of the study population. These findings align with previous studies in other general population samples than European, particularly in a population of 764 US adults, and 2230 Chinese undergraduates and 293 MDD patients [10], [20]. However, discrepancies exist in the literature regarding age and sex differences in CD-RISC-10 scores across various populations [23], [28], warranting further investigation. Socioeconomic factors also were associated with CD-RISC-10 scores, with higher average scores observed in higher income groups across our three cohorts, as well as an increase with higher educational level in SAGA and C19-Resilience. These findings support previous reports, such as Campbell-Sills, Forde, and Stein [10], who found that sex, income level, and education level demonstrated similar associations with CD-RISC-10 scores [10]. In addition, we observed a higher average CD-RISC-10 score among individuals who were in a relationship when answering the questionnaire. This could be speculated to a related strengths making it easier for high-resilient individuals to overcome difficulties and maintain stable relationships despite obstacles.

Results of our study demonstrated a positive correlation in CD-RISC-10 scores across time, with scores remaining stable for up to three years. This long-term stability supports and extends previous reports of good test-retest reliability over shorter intervals. For instance, in a Chinese cohort aged 20–63, a test-retest correlation coefficient of 0.90 was observed [22]. Long-term stability in assessing resilience suggests that the construct is a reliable and enduring characteristic that can be effectively tracked and studied over time.

We found that CD-RISC-10 scores moderated the association between self-reported financial trouble and severity of depressive symptoms. Although temporal/causal associations cannot be ascertained using cross-sectional data, this finding suggests that greater perceived resilience, as measured by the CD-RISC-10, may protect against developing depressive symptoms among individuals exposed to financial stressors. This finding aligns with the strong predictive validity of the CD-RISC observed by Velickovic et al. (2020) in a Swedish population, where CD-RISC-25 scores significantly predicted both physical and mental health-related quality of life, independent of several demographic and health variables [29].

An EFA of the CD-RISC-10 yielded a one-factor solution explaining 44–49 % of the total variance in the three cohorts, comparable to findings in other populations [21]. While this indicates that the factor captures a substantial portion of the construct, it also suggests that a considerable amount of variance remains unexplained. This could be due to the complex nature of psychological resilience, limited number of items, item-specific variance, or random response fluctuations. Future research directions could explore additional factors accounting for the unexplained variance, examine other dimensions of resilience not captured by the CD-RISC-10, and investigate how different populations or contexts might influence the factor structure. Comparative studies of various resilience scales (e.g., ‘Dispositional Resilience (Hardiness) Scale’ [30], ‘The Resiliency Attitudes and Skills Profile’ [31], and ‘The Resilience Scale’ [32]) could help identify the most comprehensive and efficient measures of psychological resilience.

This study has several notable strengths. First, it benefits from a large sample size across three cohorts. Second, the inclusion of both Danish and Icelandic populations allows for cross-cultural comparison within Nordic countries. Third, the availability of longitudinal data from the DBDS cohort enables assessment of longitudinal stability over an extended period. Fourth, the comprehensive assessment of construct validity through the examination of financial trouble's impact on depressive symptoms provides meaningful insights into the scale's practical utility. However, several limitations should be considered when interpreting our findings. First, while our sample size is large, participants were predominantly from specific population groups (blood donors in DBDS, women in SAGA, and active volunteers in C19-Resilience), which may limit generalizability to the broader population. Second, the voluntary nature of participation, particularly in the DBDS and C19-Resilience cohorts, might have led to selection bias, potentially overrepresenting individuals with higher psychological resilience. Third, our assessment of financial troubles relied on self-reported measures that, while similar, were not identical across cohorts, which could introduce measurement variability. Fourth, the cross-sectional nature of our construct validity analysis limits our ability to make causal inferences about the relationship between resilience, financial troubles, and depressive symptoms. Finally, while we observed good psychometric properties in both Danish and Icelandic versions of the CD-RISC-10, potential linguistic and cultural nuances in the translation might affect the interpretation of specific items. These nuances could influence responses, despite the overall cultural similarities between the Nordic countries. On the other hand, the cultural similarities result in additional cross-cultural studies being necessary to confirm general usability of this tool.

In conclusion, the take-home message from this study is that the CD-RISC-10 demonstrates broad utility for measurement of perceived psychological resilience across diverse populations. We recommend using this scale as a unidimensional tool by calculating the overall score based on all 10 items. The scale’s stability over time, predictive validity, and consistent performance across different cultural contexts and languages underscore it’s value as a reliable and valid tool for assessing resilience in both research and clinical settings. Its integration into mental health interventions can provide insights into resilience trajectories and support the development of targeted resilience-building strategies. Future research should aim to validate these findings in diverse global populations and explore the scale’s utility in predicting long-term health outcomes.

## Ethics

For DBDS, participants provided written informed consent and the study was approved by the Scientific Ethics Committee (1−10−72−95−13). The participants of SAGA and C-19 Resilience cohorts provided electronic informed consent with an e-Identification and both studies were approved by the National Bioethics committees (17–238 and 20–073, respectively).

## Declaration of Competing Interest

The authors declare that they have no known competing financial interests or personal relationships that could have appeared to influence the work reported in this paper.
