# Supplementary material for: Psychometric properties and socio-demographic correlates of the Connor-Davidson Resilience Scale in three large population-based cohorts including Danish and Icelandic adults
Source: J Mood Anxiety Disord. 2025 Feb 17;10:100112. doi: 10.1016/j.xjmad.2025.100112 (PMC12244040; doi:10.1016/j.xjmad.2025.100112)
Supplement: Supplementary file 1 — Supplementary material [file mmc1.docx]

# **Figure S1**: Sex-stratified parallel scree plots

**
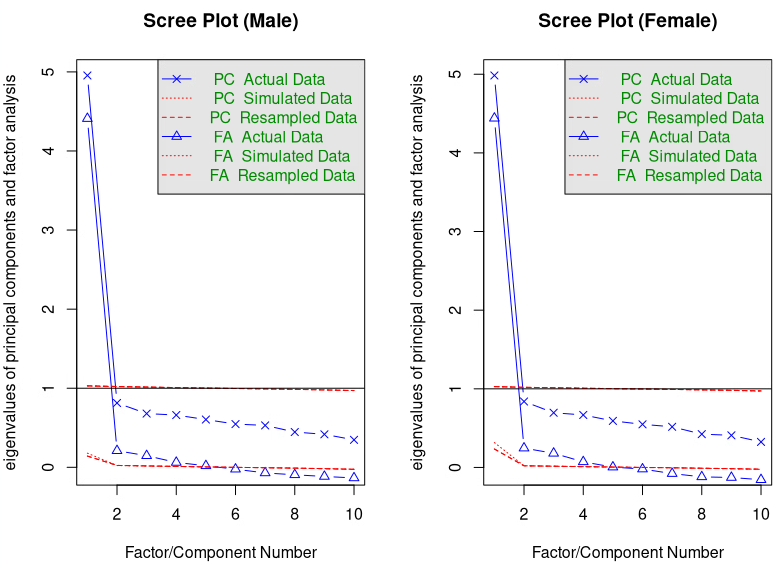
**DBDS:

C19-Resilience:


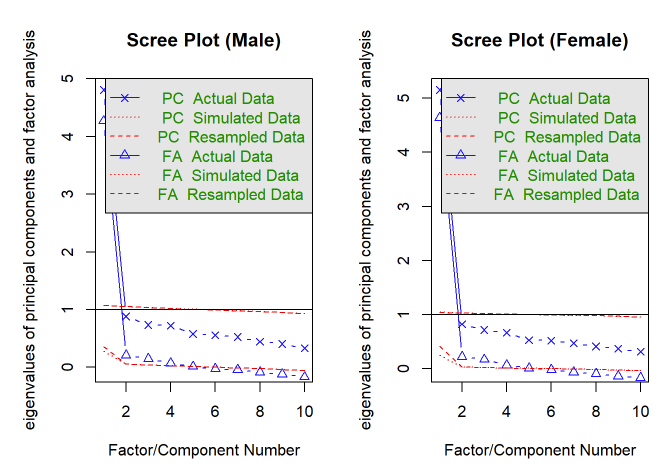


# **Figure S2.** Effect of CD-RISC-10 scores in moderating the association between financial trouble and PHQ-9 scores in women and men


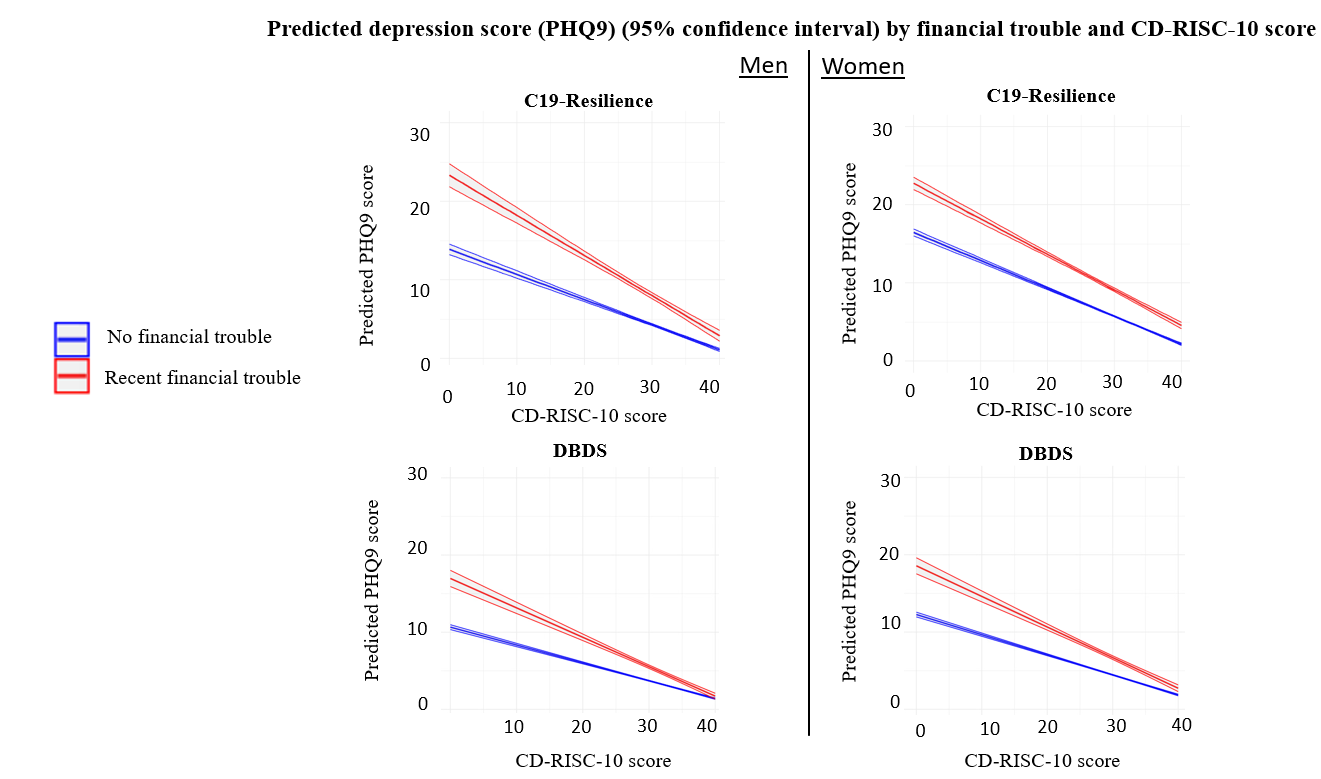


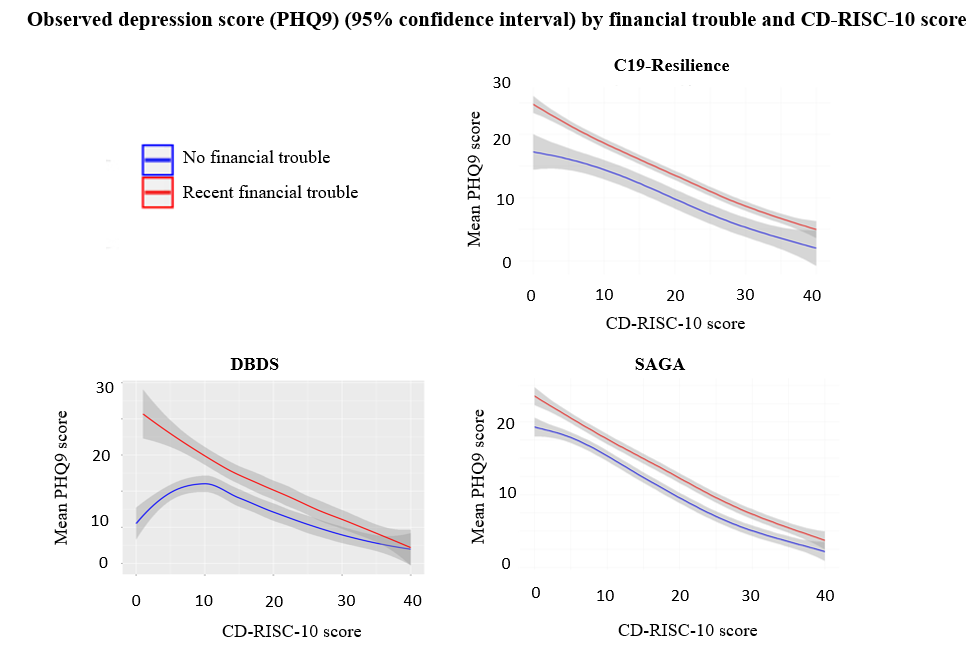
**Figure S3.** Displaying observed PHQ9 and CD-RISC-10 scores stratified by experience of recent financial troubles


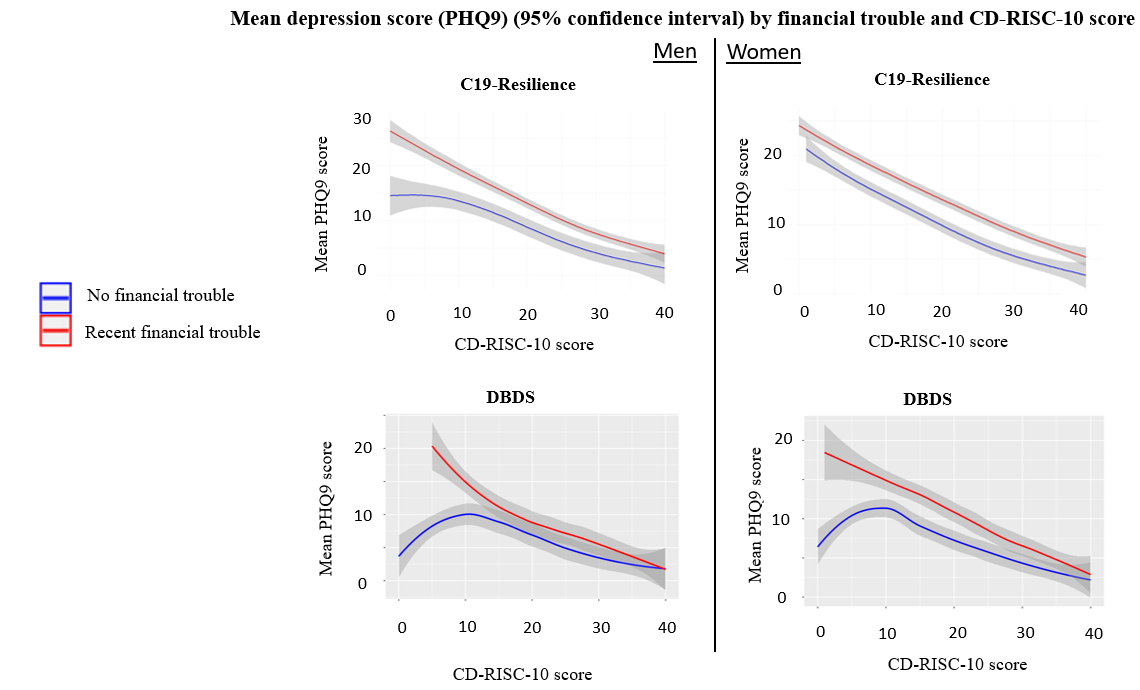
**Figure S3.** Displaying observed PHQ9 and CD-RISC-10 scores stratified by experience of recent financial troubles
